# Low-dose aspirin for primary and secondary prevention of cardiovascular events in Denmark 1998–2018

**DOI:** 10.1038/s41598-021-93179-8

**Published:** 2021-06-30

**Authors:** Mikkel B. Christensen, Espen Jimenez-Solem, Martin. T. Ernst, Morten Schmidt, Anton Pottegård, Erik L. Grove

**Affiliations:** 1 Department of Clinical Pharmacology, Bispebjerg Hospital, University of Copenhagen, Bispebjerg Bakke 23, 2400 Copenhagen, Denmark; 2 Department of Clinical Medicine, University of Copenhagen, 2100 Copenhagen, Denmark; 3 Copenhagen Center for Translational Research, Bispebjerg Hospital, University of Copenhagen, 2400 Copenhagen, Denmark; 4 Clinical Pharmacology and Pharmacy, Department of Public Health, University of Southern Denmark, 5000 Odense, Denmark; 5 Department of Cardiology, Aarhus University Hospital, 8200 Aarhus, Denmark; 6 Department of Clinical Epidemiology, Aarhus University Hospital, 8200 Aarhus, Denmark; 7 Department of Clinical Medicine, Faculty of Health, Aarhus University, 8200 Aarhus, Denmark

**Keywords:** Epidemiology, Cardiology

## Abstract

Randomised controlled trials have shown a neutral or even unfavourable risk–benefit balance of aspirin for primary prevention of cardiovascular events. Using Danish nationwide registries, we investigated aspirin use and associated risks during the past two decades (1998–2018). We linked individual patient data on repeated aspirin redemptions with registered hospital ICD-10 diagnoses of atherosclerotic cardiovascular disease and bleedings. The prevalence of aspirin use among 1.1 million Danish adults fluctuated over the 20-year study period peaking in 2008 with 8.5% (5.4% primary prevention) and dropping to 5.1% (3.1% primary prevention) in 2018. Aspirin use showed strong age dependency, and 21% of individuals > 80 years were treated with aspirin for primary prevention in 2018. Medication adding to bleeding risk was used concurrently by 21% of all aspirin users in 2018. The incidence of major bleedings were similar with primary and secondary prevention aspirin use and highest in elderly (2 per 100 patient years among individuals > 80 years in 2018). In conclusion, low-dose aspirin use for primary prevention of cardiovascular events remains prevalent. The widespread use of aspirin, especially among older adults, and substantial concomitant use of medications adding to bleeding risk warrant increased focus on discontinuation of inappropriate aspirin use.

## Introduction

Aspirin is a well-established and inexpensive antiplatelet drug used to prevent cardiovascular events in patients with atherosclerotic cardiovascular disease (ASCVD)^1^. While a favourable risk–benefit balance is well-established for low-dose (75–150 mg) aspirin use in patients with ASCVD^2^, the net clinical benefit of primary aspirin treatment in patients without established ASCVD is less clear^3–5^. Thirty years ago, the first clinical trials reported a potential cardiovascular protective effect of relatively high-dose (500 mg) aspirin in individuals without ASCVD^6,7^. Since then, several randomized clinical trials have investigated the risk–benefit profile of primary prevention of cardiovascular events with low-to moderate dose (75–325 mg) aspirin in patients without established ASCVD^3,4^. Based on accruing evidence throughout the 2000s, international guidelines recommended primary prevention aspirin in adults with conventional risk factors for ASCVD. Around 2010 guideline directions for the primary prevention use of aspirin increasingly had diverged^8^, and several therapeutic antithrombotic alternatives to aspirin for ASCVD and other conditions with heightened thrombotic risk had emerged^9,10^. In 2016 the European Society of Cardiology unilaterally decided to recommend against aspirin in patients without overt ASCVD^11^. In 2018, clinical trials investigated the effect of primary prevention aspirin in healthy elderly (ASPREE)^12–14^, patients with diabetes (ASCEND)^15^, and patients with multiple risk factors for CV disease excluding patients with diabetes (ARRIVE)^16^. The trial data consistently supported that primary prevention with aspirin in general should be avoided as small absolute ASCVD benefits were exceeded or balanced by bleeding hazards. Moreover, the largest trial did not support any preventive effect of aspirin on cancer incidences in older adults^17^. In 2019 the American Heart Association and American College of Cardiology (AHA/ACC) adopted a recommendation against the routine use of aspirin for primary cardiovascular prevention, particularly in individuals older than 70 years or patients with increased risk of bleeding^18^.

The historical uncertainty regarding primary prevention aspirin use may have led to what in retrospect could be perceived as overtreatment. To clarify the extent of such potentially inappropriate aspirin use—before and now—we performed a nationwide drug utilization study exploring aspirin use for primary prevention during the past two decades (1998–2018).

## Methods

### Data sources

We linked data from Danish registries using the Civil Personal Register number, a unique identifier assigned to all Danish residents since 1968^19^. From the Danish Prescription Registry^20^, we obtained data on all prescription drugs (ATC-code, quantity, and date of dispensing). From the Civil Registration System^21^, we obtained data on death and migration to and from Denmark. From the Danish National Patient Register^22^, we obtained data on all diagnoses according to ICD-10 (International Classification of Diseases, 10th revision). We defined ASCVD as those with a diagnosis of myocardial infarction, stable or unstable angina, coronary revascularization, peripheral artery disease, ischaemic stroke, or transient ischaemic attack (see supplementary Table 1 for ICD codes).

### Population and study drugs

We included all adults (> 18 years) redeeming a prescription for low-dose aspirin in strengths ≤ 150 mg (ATC codes B01AC06 and N02BA01). In Denmark, higher doses of aspirin are not used for prevention of CV events. For several analyses, we had to estimate whether an individual was a ‘current user’ of aspirin at a given date. We considered an individual as a ‘current user’ if they had recently filled a prescription for aspirin with enough tablets to cover that day. The duration of each prescription was estimated as the number of tablets dispensed, that is assuming a consumption of one tablet per day, while adding 20% to the duration to account for noncompliance and irregular prescription refills.

### Analyses

First, we calculated the annual rate of new users per 1000 adults in the population (as an estimate of incidence) and proportion of total users (point prevalence) using the total Danish adult population on 1 January of the relevant year as the denominator. New users were defined as individuals without low-dose aspirin prescription use in a preceding 5-year period or since the establishment of the Danish National Prescription Database in 1995. Aspirin use was divided into primary and secondary prevention based on cardiovascular diagnoses at the time of redeeming the prescription. Further, the sex and age-specific (using one-year intervals) prevalence proportion was reported each 10 years (1998, 2008 and 2018). Second, we described treatment duration, using the ‘proportion of patients covered’ method^23^. In brief, we followed all individuals from the date of their first aspirin prescription fill. Over time, we estimated the proportion of all subjects still alive after X days and still using aspirin at that day. As such, an individual could be regarded as ‘dropped out of treatment’ at one point in time and later be reclassified as ‘current user’ upon redeeming a new prescription. Subgroup analyses were carried out according to age, sex, and calendar year of first prescription. Third, we identified the proportion of individuals who, after their first aspirin prescription, filled at least one prescription for aspirin each year for 5 consecutive years (defined as years from first prescription). Fourth, in order to explore potential bleeding risk in relation to aspirin use, we estimated the prevalence of concomitant use (≥ 1 prescription) of co-medication known to affect the risk of gastrointestinal bleeding among users of aspirin (increase risk of bleeding: Non-steroidal anti-inflammatory drugs (NSAID), serotonin reuptake inhibitors (SSRI), other antiplatelet drugs, oral anticoagulants, and systemic glucocorticoids; lower risk of bleeding: Proton pump inhibitors (PPI)) standardised to the age-distribution of 2018. Only prescriptions redeemed by individuals currently using aspirin or during the 30 days leading up to aspirin use were included. As a sensitivity analysis, we also analysed this using a 90-day look back period (which did not change the estimates substantially). Fifth, we estimated age-standardised annual bleeding rate by including established hospital diagnoses of bleeding (see Supplementary Table 1). Data was analysed using Stata Version 16.1 (StataCorp, College Station, TX, USA).

### Ethics

According to Danish law, anonymized studies based solely on registry data do not require approval from an ethics review board. In terms of data protection, the study was registered at the University of Southern Denmark’s inventory (record no. 10.446).

### Results

We identified 1.1 million adult aspirin users during the 21-year study period, during which the adult Danish population increased from 4.19 to 4.62 million individuals. Among users, 161,022 (14%) individuals redeemed only one prescription, whereas 147,659 (13%) redeemed two to four, and 818,484 (73%) individuals redeemed ≥ five prescriptions.

**Figure 1:**
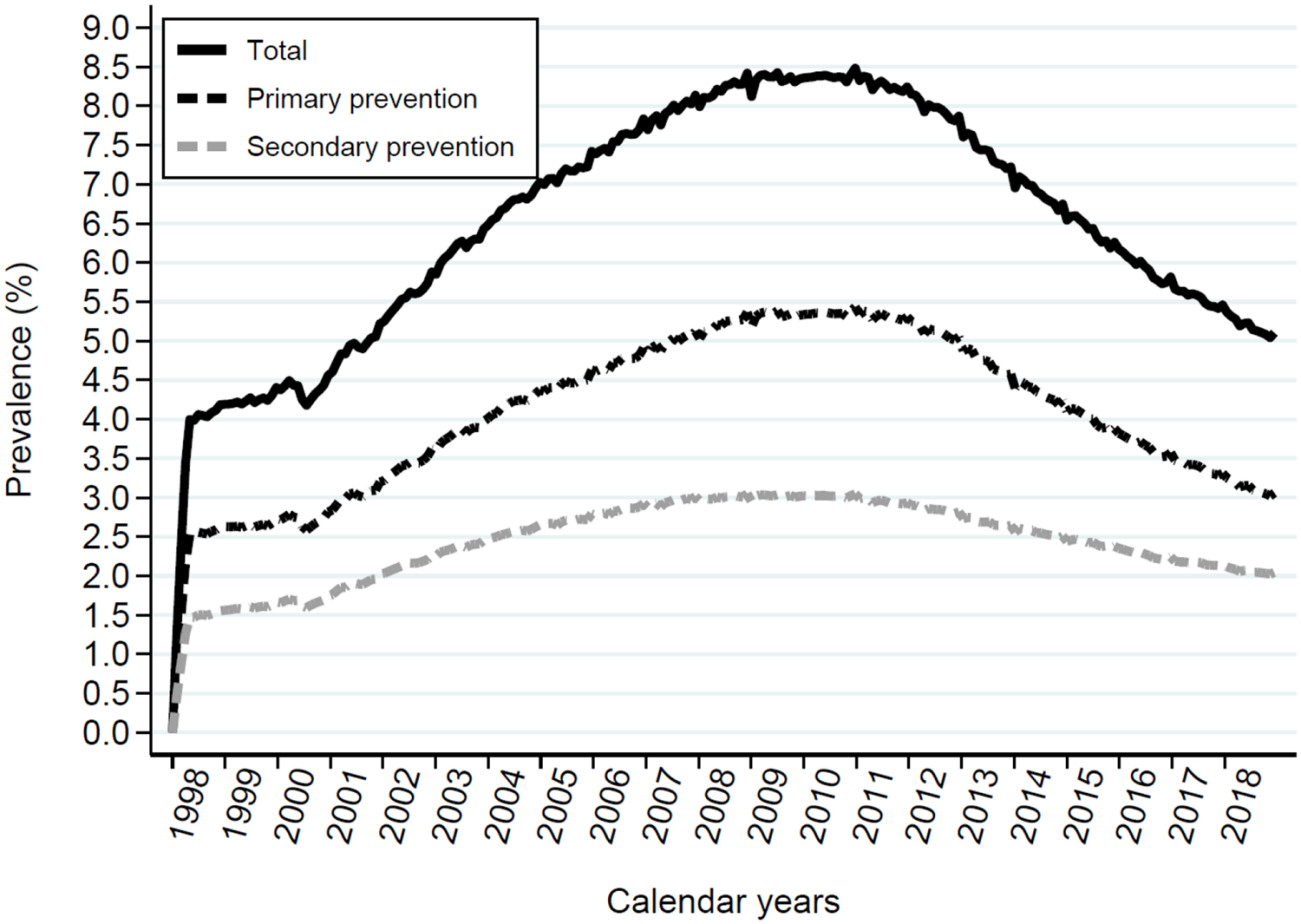
Panel (**A**) shows Aspirin use expressed as total point prevalence among the adult population in Denmark within a 30-day time period (black line) and separated into primary prevention (black dotted line) and secondary prevention (grey dotted line). Panel shows the distribution of aspiring use in.

The prevalence of aspirin use followed a unimodal curve over the study period (Fig. 1). The prevalence of aspirin users was 4.2% in 1998, 8.5% in 2008, and 5.1% in 2018. Primary prevention use amounted to 2.6% in 1998, 5.4% in 2008, and 3.1% in 2018, accounting for 62%, 64, and 61% of all prescribed low-dose aspirin the respective years.

**Figure 2:**
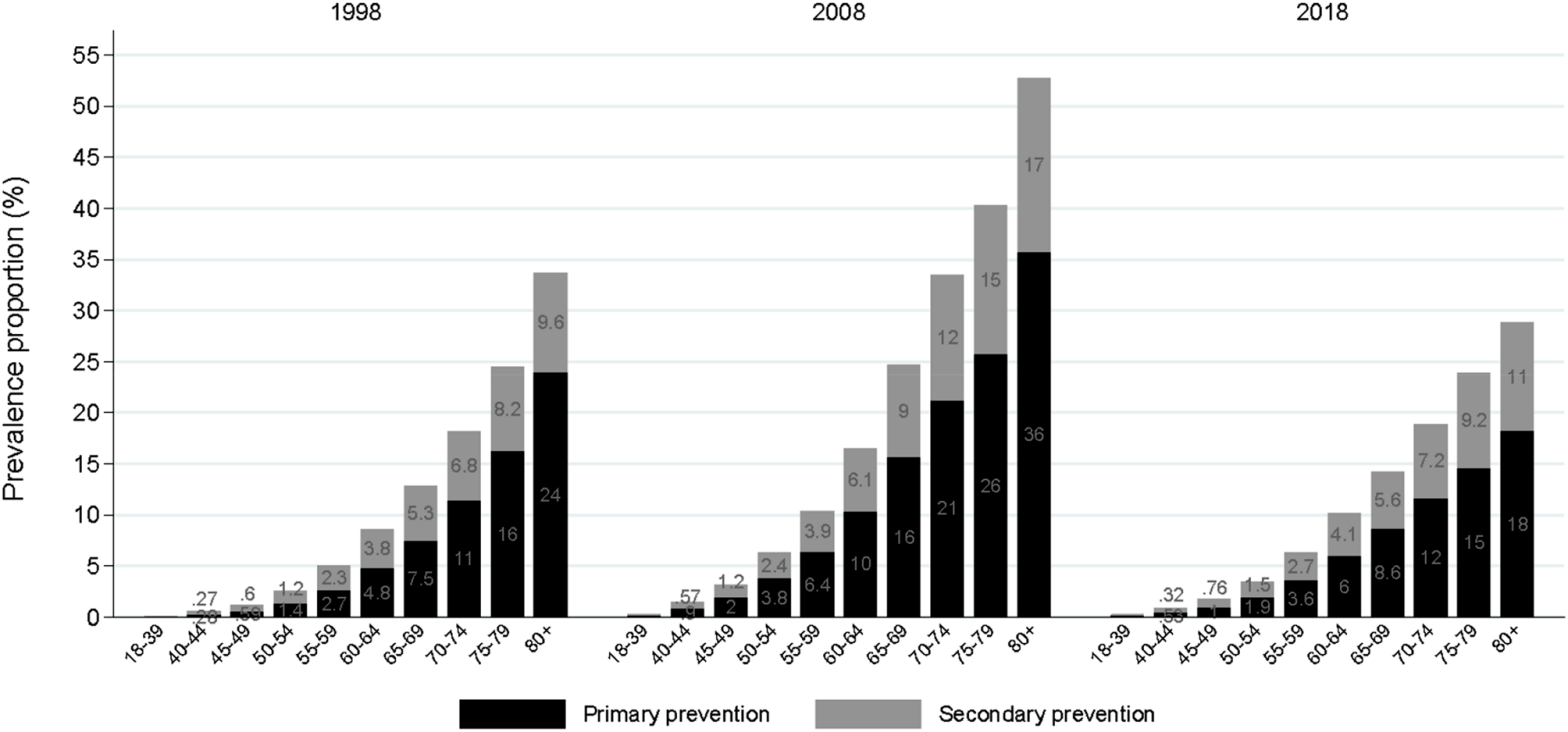
Prevalence of aspirin use stratified by age groups and primary vs. secondary prevention in 1998, 2008 and 2018. The age continuum for aspirin users divided into primary prevention (black bars) and secondary prevention (grey bars) use at selected years in the study period.

Figure 2 depicts the age continuum for prevalence of aspirin use for selected years in the study period. There was a general, steadily increasing use of aspirin for both primary and secondary with increasing age. The prevalence of aspirin use for primary prevention among those aged at least 80 years was approximately 27% in 1998, 40% in 2008, and 20% in 2018.

**Figure 3:**
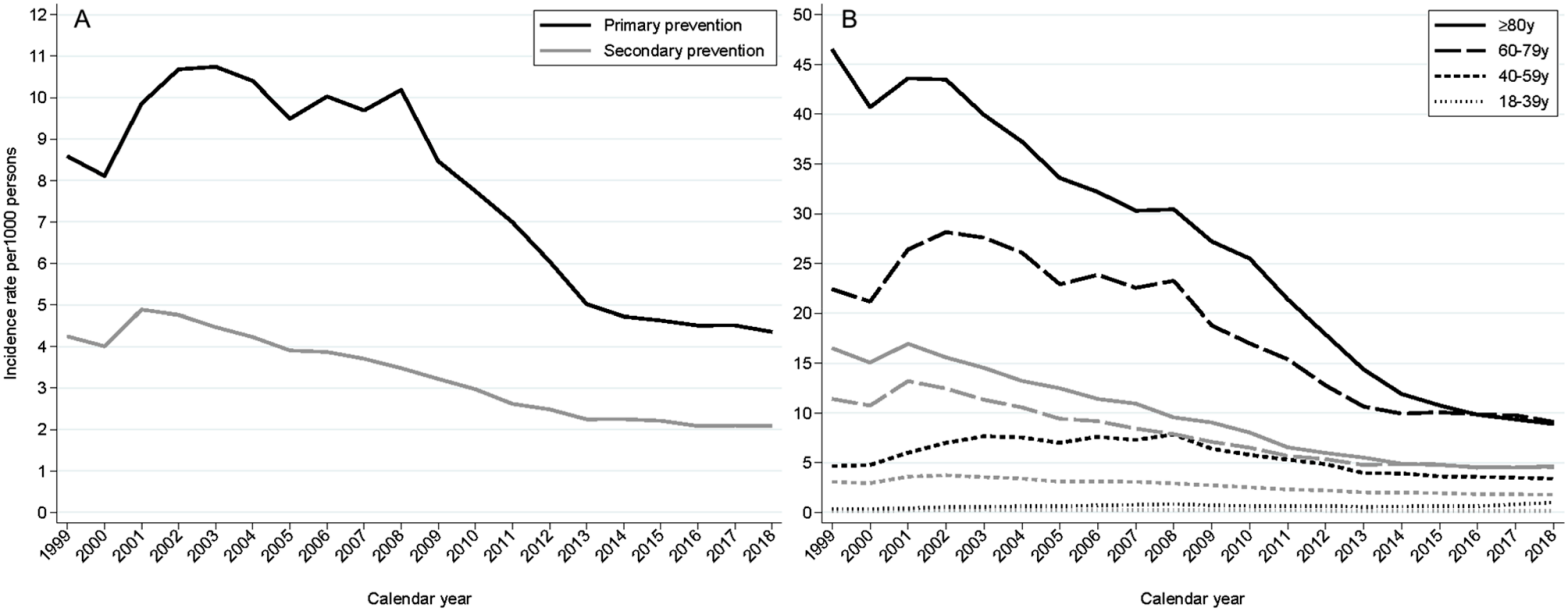
Incidence rate of new aspirin users stratified by primary and secondary prevention and age groups. The incidence of new users redeeming their first of at least two prescriptions divided into primary (black lines) and secondary prevention (grey lines) for the entire adult population (Panel **A**), and age intervals for primary (black lines) and secondary prevention (grey lines) (Panel **B**).

The incidence rate was independent of whether aspirin was used for primary or secondary prevention (Fig. 3A). While the proportion of incident users who used aspirin for secondary prevention was relatively stable over time, the incidence rate for primary prevention declined after 2008 from 11 to 4 incident users per 1000 person-years (Fig. 3A). The incidence rate was age-dependent and highest in patients older than 80 years to 2015 and thereafter was equivalent to people aged 60–80 years (Fig. 3B).

**Figure 4:**
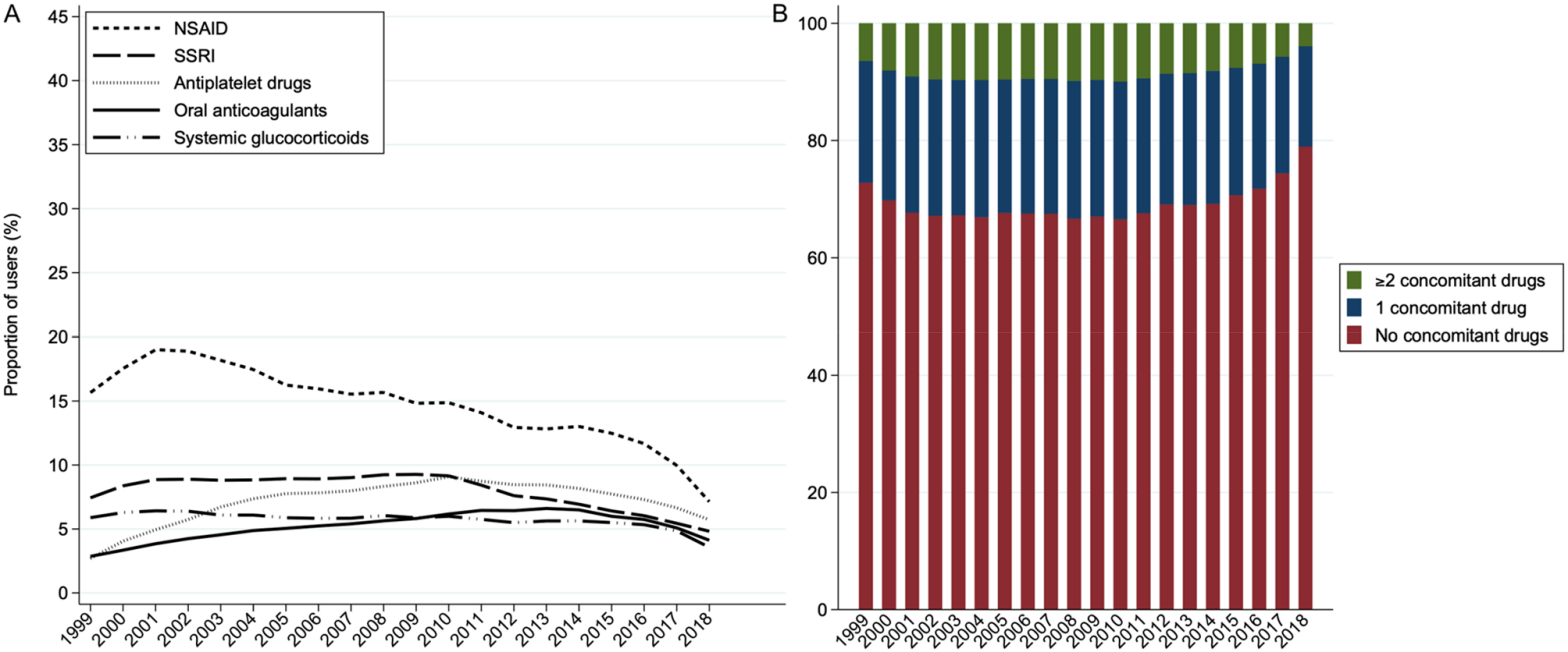
Primary prevention use of aspirin and concomitant use of drugs known to increase the risk of gastrointestinal bleeding. The prevalence of concomitant use of drugs known to affect the risk of gastrointestinal bleeding (Panel **A**) divided into non-steroidal anti-inflammatory drugs (NSAID), serotonin reuptake inhibitors (SSRI), other antiplatelet drugs, oral anticoagulants, systemic glucocorticoids and proton pump inhibitors (PPI) among primary prevention aspirin users. Panel (**B**) shows the percentage of aspirin users treated simultaneously with either none, one or more than one drug, respectively, known to increase the risk of bleeding.

The proportion of aspirin users redeeming a concomitant prescription of at least one drug known to affect the risk of gastrointestinal bleeding differed slightly between primary and secondary aspirin users (see Fig. 4 and Supplementary Fig A). Among primary prevention aspirin users, the concomitant use of a drug class known to increase bleeding (NSAID, SSRIs, other platelet inhibitors, systemic glucocorticoids and oral anticoagulants) was highest in 2002–2010 with approximately 33%, and in 2018 it had dropped to its lowest prevalence of 21% (Fig. 4). The largest change among primary prevention aspirin users was for NSAID, which had a prevalence of 19% in 2001–2002 that had dropped to 7.1% in 2018 (Fig. 4A). Use of SSRIs, other platelet inhibitors, systemic glucocorticoids and oral anticoagulants fluctuated slightly over the study period, and in 2018 among primary prevention aspirin the proportions of concomitant users for these drug classes were 4.8%, 5.7%, 3.6% and 4.1%, respectively. Use of PPI in primary prevention aspirin users almost doubled over the study period from 8.6% in 1999 to 16% in 2018.

**Figure 5:**
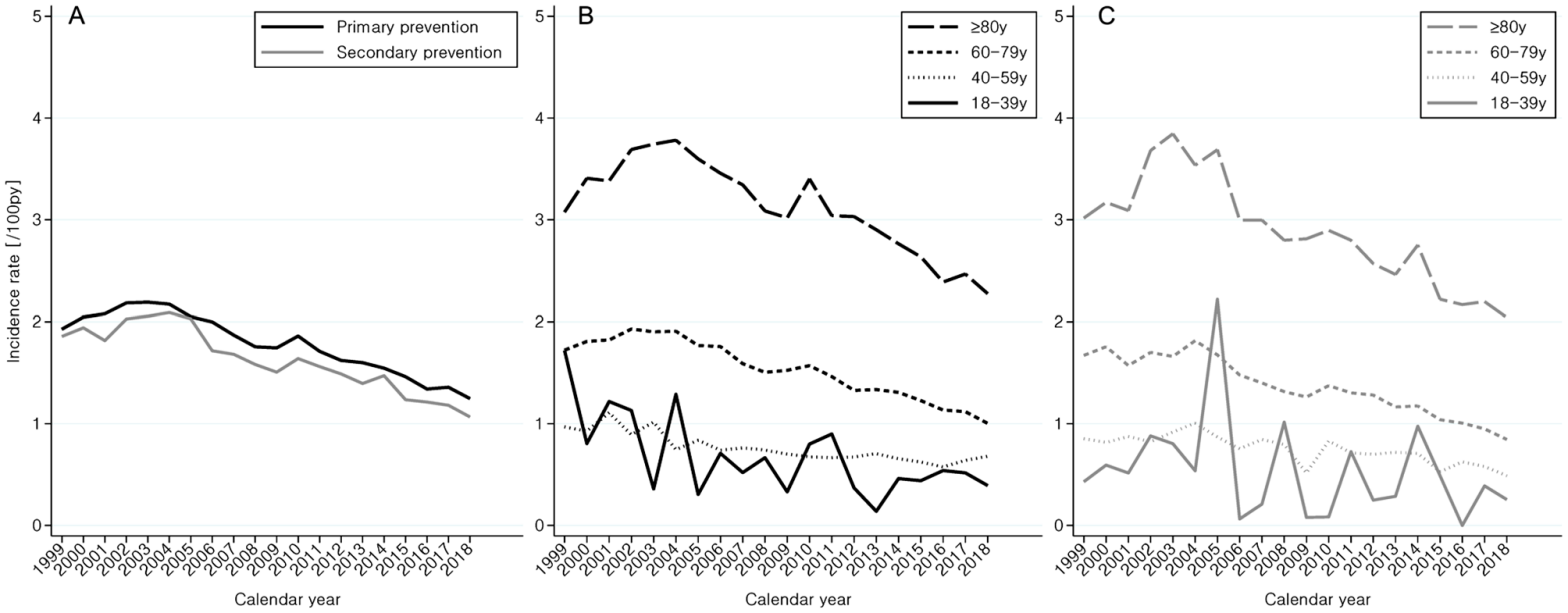
Incident bleeding events in aspirin users. The incidence of major bleedings with primary prevention aspirin use (black lines) or secondary prevention aspirin use (grey lines) for the entire population (Panel **A**) of, or according to age intervals as either primary prevention (Panel **B**) use or secondary prevention (panel **C**) and age-standardised to 2018.

The incidence of major bleedings in aspirin users was similar with primary and secondary prevention use and has been declining since 2002 (Fig. 5). The bleeding incidence was higher with increasing age, and in 2018 it amounted to 2.2 per 100 patient years among primary prevention aspirin users aged 80 years or more.

Persistence to treatment estimated by proportion of patients covered (Supplementary Fig B) was generally lower with aspirin used for primary prevention than for secondary prevention. Patients who initiated aspirin therapy between 2002–2005 had a higher treatment persistence than patients who initiated aspirin in 2006–2012 or from 2013–2018 (Supplementary Fig B). In general, patients older than 60 years had a high treatment persistence, with almost 60% of both primary and secondary prevention aspirin users redeeming prescription for at least 5 years (Supplementary Fig B).

## Discussion

In 2018, low dose-aspirin was still more frequently prescribed for primary than secondary prevention. A total of 3.6% of the entire adult population used aspirin without a registered diagnosis of ASCVD, whereas more than 20% of Danish citizens aged 80 years or older were treated with aspirin for primary prevention, and this group also had a substantial annual hospital-diagnosed bleeding risk of 2.2%.

### Strength and limitations

A major strength of our study is the nationwide capture of prescription redemptions and relevant diagnosis from national registers. However, a limitation of these data is that we were unable to include data related to non-prescription aspirin use (i.e. related to over the counter (OTC) sale) of aspirin. In Denmark, non-prescription OTC-sale is restricted to pharmacies and amounted to about 10% of the total low-dose aspirin sale in 2006–2018 (available from medstat.dk^24^). However, as prescription sale of aspirin is reimbursed in Denmark, it is plausible that OTC sale mostly was for people without a prescription (i.e. in most cases not based on an established ASCVD diagnosis). Therefore, the fact that we did not have access to individual non-prescription sale of aspirin most likely leads to an underestimation of the primary prevention use of aspirin in the Danish population. Another issue is misclassification of primary vs. secondary prevention, since patients only treated in primary care may not have a central registration of diagnoses for ASCVD and/or bleeding, which potentially could lead to some underestimation of ASCVD and bleeding diagnoses in our dataset. However, as manifestations of ASCVD are generally symptomatic and treated in specialized secondary care facilities, where the diagnoses are also established, substantial misclassification for ASCVD is not likely. The high accuracy of cardiovascular ICD diagnoses in Denmark support this notion^25^. In contrast, we have likely underestimated harms in terms of minor bleeding events, as only severe bleeding events necessitate in-hospital management.

### Previous literature

There are few other contemporary studies investigating population-based aspirin use. In a recent cohort from the 2017 US National Health Interview Survey (NHIS) (n = 14,328), self-reported aspirin use ranged from 7% among individuals aged 40–50 years to 46% among individuals aged 80 years or more^26^. In contrast to our registry data, the NHIS defined primary prevention aspirin use as no self-reported angina, coronary heart disease, myocardial infarction, or stroke. The 2017 US prevalence numbers are much higher than the corresponding numbers in our cohort, where less than 2% of the individuals aged 40–50 years and 20% of individuals aged 80 years or more were taking aspirin in 2017. This difference may in part be due to unregistered OTC use in Denmark in particular the early periods of our study periods, but even more likely due to differences in prescription patterns. Thus, as previously mentioned opinions on the risk–benefit balance of aspirin differed between Europe and the US during the 2010s. In Denmark, the prevalence of aspirin use for primary prevention among citizens aged 80 years peaked at 36% in 2010, and was reduced to almost half of that by 2018. The lower prevalence of aspirin use in our data compared to NHIS data may also reflect differences between registry data and self-reported diagnoses and aspirin use, notably there was final response rate of only 53% in the 2017 NHIS^26^. Our registry-based estimates relying on prescription data and physician-diagnosed ASCVD based on ICD codes include the whole population are probably more accurate and conservative, which result in lower prevalence estimates.

Our findings and the US NHIS data support that overtreatment with aspirin occurred during the past decades and, based on 2018 data, still occurs. A recent meta-analysis including populations with and without diabetes concludes that the benefit of aspirin for primary prevention in terms of preventing major cardiovascular events is a relative risk reduction of 11% (95% CI 6–16%), which has to be compared to a 43% relative risk increase of major bleeding events (95% CI 30–56%)^27^.

Collectively, the large extent of primary prevention aspirin use in light of the updated evidence suggest that a substantial amount of the treatment is potentially inappropriate and could be discontinued, preferably based on shared decision making between prescriber and patient^28^. However, the decision on whether to initiate, continue or discontinue aspirin for primary prevention in and by individual patients can be challenging. As corroborated by our data, increasing age generally leads to increased bleeding risk. Thus, the current US guidelines discourage routine aspirin use in patients over 70 years, but offer little help in deciding which older patients (if any) without established ASCVD, who should be treated with aspirin. Patients with type 2 diabetes is another large and challenging patient group. European and U.S. guidelines has historically offered divergent interpretations of the available clinical trial evidence in these patients^29^. The recent ASCEND trial^15^ with a mean follow-up of 7.4 years showed that in patients with type 2 diabetes, an absolute 1.1%-point reduction of serious vascular events (8.5% with aspirin treatment vs 9.6% with placebo) was balanced by a 0.9%-point excess of major bleeding events (4.1% with aspirin treatment vs 3.2% with placebo). Notably, the most favourable risk–benefit balance was observed in individuals with the lowest vascular risk score (baseline 5-year risk of serious vascular events < 5%). In contrast, among the subgroup with the highest risk of ASCVD (baseline 5-year risk of serious vascular events ≥ 10%), the avoided number of deaths from cardiovascular causes was numerically lower than the increase in fatal bleeding events^15^. The most recent European and US guidelines on the use of antiplatelet agents for patients with type 2 diabetes recommends that primary prevention use of low dose aspirin may be considered ‘in patients at high/very high cardiovascular risk, in the absence of clear contraindications’ or ‘in those, who are at increased cardiovascular risk, after a discussion with the patient on the benefits versus increased risk of bleeding’^30,31^. Thus, there seems to be a great need for clear, supportive guidelines and easy understandable information to help clinicians and patients balancing the risks and benefits of aspirin for primary prevention based on individual patient characteristics.

## Conclusion

Low-dose aspirin use for primary prevention of ASCVD is prevalent and increases with age. In 2018, it was still more prevalent than secondary prevention use. The widespread use of aspirin, especially among older adults, and substantial concomitant use of medications adding to bleeding risk warrants increased focus on discontinuation of inappropriate aspirin use.

## Supplementary Information

Supplementary Information.

## Data Availability

The data that support the findings of this study are available from Statistics Denmark and restrictions apply to the availability of these data, which were used under license for the current study and therefore are not publicly available. Data are however available from the authors upon reasonable request and with permission of Statistics Denmark.

## Abbreviations

ASCVD: Atherosclerotic cardiovascular disease
NHIS: US National health interview survey
AHA/ACC: American Heart Association and American College of Cardiology
ATC: Anatomical therapeutic chemical classification
CI: Confidence interval
ICD-10: International classification of diseases, 10th revision
NSAID: Nonsteroidal antiinflammatory drugs
OTC: Over the counter
