# Supplementary material for: Low-dose aspirin for primary and secondary prevention of cardiovascular events in Denmark 1998–2018
Source: Sci Rep. 2021 Jun 30;11:13603. doi: 10.1038/s41598-021-93179-8 (PMC8245534; doi:10.1038/s41598-021-93179-8)
Supplement: Supplementary file 1 — Supplementary Information. [file 41598_2021_93179_MOESM1_ESM.docx]

**Low-dose aspirin for primary and secondary prevention of cardiovascular events in Denmark 1998-2018**

Mikkel B. Christensen^1,2,3^, Associate professor (0000-0002-8774-1797); Espen Jimenez-Solem^1,2^, Associate professor; Martin. T. Ernst^4^, MSc; Morten Schmidt^5,6^, Associate professor; Anton Pottegård^4^, professor; Erik L. Grove^5,7^, Associate professor (0000-0002-1466-0865).

^1^Department of Clinical Pharmacology, Bispebjerg Hospital, University of Copenhagen, DK-2400 Copenhagen, Denmark

^2^Department of Clinical Medicine, University of Copenhagen, DK-2100 Copenhagen, Denmark

^3^Copenhagen Center for Translational Research, Bispebjerg Hospital, University of Copenhagen, DK-2400 Copenhagen, Denmark

^4^Clinical Pharmacology and Pharmacy, Department of Public Health, University of Southern Denmark, DK-5000, Odense, Denmark

^5^Department of Cardiology, Aarhus University Hospital, DK-8200, Aarhus, Denmark

^6^Department of Clinical Epidemiology, Aarhus University Hospital, DK-8200, Aarhus, Denmark

^7^Department of Clinical Medicine, Faculty of Health, Aarhus University, DK-8200, Aarhus, Denmark

**Supplementary figures and table**

**Supplementary Figure A. Secondary prevention use of aspirin and concomitant use of drugs known to increase the risk of gastrointestinal bleeding**


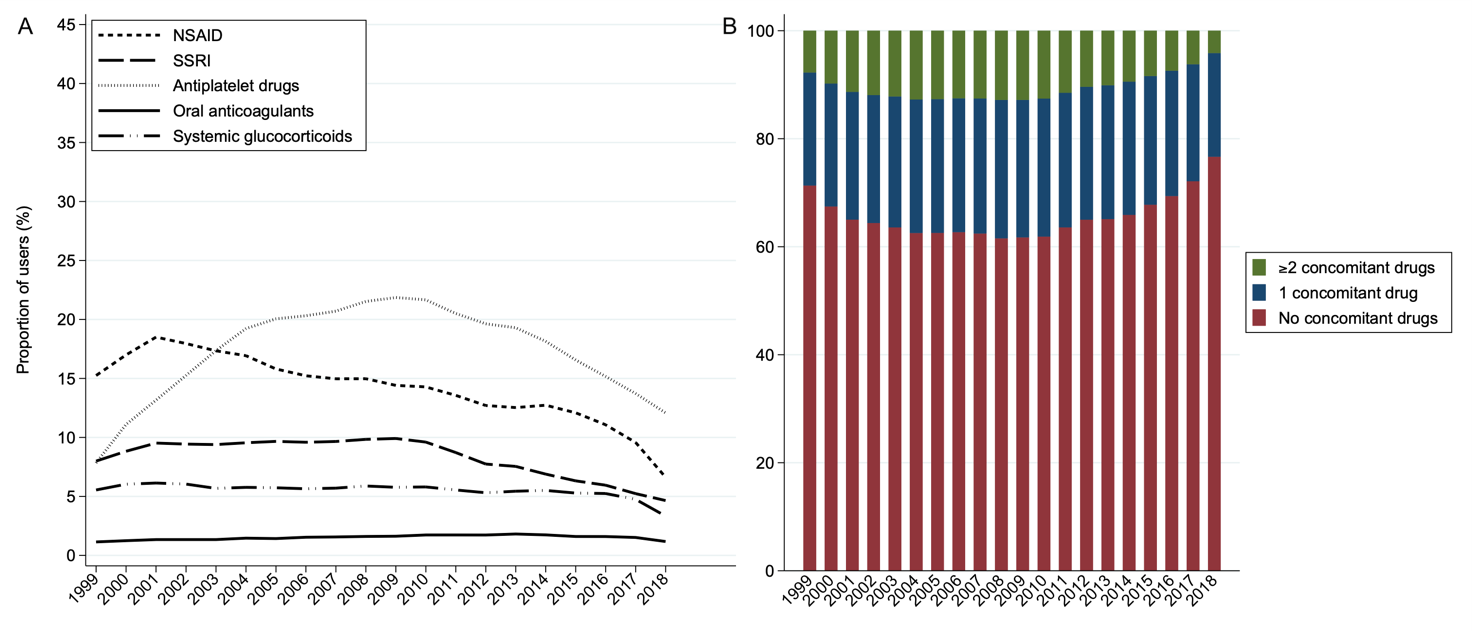


**Supplementary Figure A**. The prevalence of concomitant use of drugs known to affect the risk of gastrointestinal bleeding (Panel A) divided into non-steroidal anti-inflammatory drugs (NSAID), serotonin reuptake inhibitors (SSRI), other antiplatelet drugs, oral anticoagulants, systemic glucocorticoids and proton pump inhibitors (PPI) among secondary prevention aspirin users. Panel B shows the percentage of aspirin users treated simultaneously with either none, one or more than one drug, respectively, known to increase the risk of bleeding.

**Supplementary Figure B. Proportion of aspirin users still using aspirin five years after initiation**

**
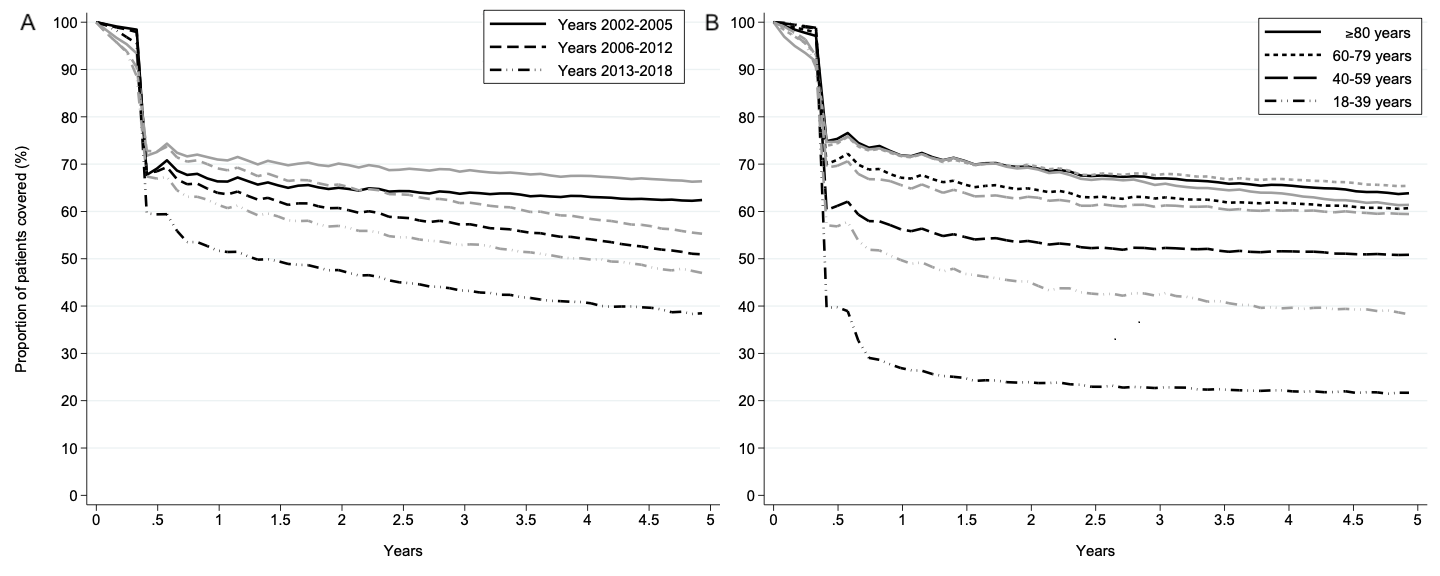
Supplementary Figure B.** Duration of aspirin treatment as primary prevention (grey lines) and secondary prevention (black lines) calculated as the ‘proportion of patients covered’ up to 5 years following each individual users first prescription (see methods section for description) stratified by time period of aspirin initiation (panel A) and age group (panel B),

**Supplementary Table 1.** ICD codes for primary and secondary atherosclerotic cardiovascular disease

| Secondary prevention | |
| --- | --- |
| Myocardial infarction | ICD8: 410  ICD10: I21 |
| Stable / unstable angina | ICD8:411 413  ICD10: I20 I251 I259 |
| Coronary revascularization | Procedure codes: KFNG KFNF KFNA KFNB KFNC KFND KFNE KFNH20 |
| Peripheral arterial disease | ICD8: 440 441 442 443 444 445  ICD10: I70 I71 I72 I73 I74 I77 |
| Ischemic stroke | ICD8: 433 434  ICD10: I63 I64 |
| Transient ischemic attack (TIA) | ICD8: 435  ICD10: G45.9 |
| Primary prevention |  |
| Diabetes | ICD8: 249.00 249.06 249.07 249.09 250.00 250.06 250.07 250.09  ICD10: E10 E11 E13 E14 H36.0  ATC: A10 |
| Atrial fibrillation and flutter | ICD8: 427.93 427.94  ICD10: I48 |
| Heart failure | ICD8: 427.09 427.10 427.11 427.19 428.99 782.49  ICD10: I50.0 I50.1 I50.2 I50.3 I50.8 I50.9 I11.0 I13.0 I13.2 I42.0 I42.6 I42.7 I42.8 I42.9 |
| Bleeding (admissions only) | ICD8: 430 431 783.09 783.19 530.98 531.90 531.92 531.95 532.90 533.90 534.90 535.01 456.01 569.15 789.39  ICD10: I60 I61 I62 R04.0 R04.2 I85.0 K25.0 K25.2 K25.4 K25.6 K26.0 K26.2 K26.4 K26.6 K27.0 K27.2 K27.4 K27.6 K28.0 K28.2 K28.4 K28.6 K29.0 K62.5 K92.0 K92.1 K92.2 R31.9 N02 D62 |
| Gastrointestinal tract (GI)  bleeding (admissions only) | ICD8: 530.98 531.90 531.92 531.95 532.90 533.90 534.90 535.01 456.01 569.15  ICD10: I85.0 K25.0 K25.2 K25.4 K25.6 K26.0 K26.2 K26.4 K26.6 K27.0 K27.2 K27.4 K27.6 K28.0 K28.2 K28.4 K28.6 K29.0 K62.5 K92.0 K92.2 |

**Supplementary Table 2**. Concomitant use of drugs known to increase the risk of gastrointestinal bleeding

|  | |
| --- | --- |
| “Bleed risk drugs” | |
| NSAIDs | M01A* (excl. M01AX*) |
| Vitamin K antagonists and oral anticoagulants | B01AA* B01AE07 B01AF01 B01AF02 B01AF03 B01AX06 |
| Non-Aspirin platelet inhibitors incl. ADP-receptor blockers | B01AC04 B01AC07 B01AC22 B01AC24 B01AC30 |
| Glucocorticoids for systemic use | H02AB |
| SSRI | N06AB |
